# Dynamic Characteristic Analysis of a Toothed Electromagnetic Spring Based on the Improved Bouc—Wen Model

**DOI:** 10.3390/ma16134889

**Published:** 2023-07-07

**Authors:** Xiaoyuan Zheng, Cheng Zhang, Yifang Lou, Guangming Xue, Hongbai Bai

**Affiliations:** 1 College of Mechanical Engineering and Automation, Fuzhou University, Fuzhou 350116, China; 2 Metal Rubber Engineering Research Center, Fuzhou University, Fuzhou 350116, China

**Keywords:** electromagnetic spring, dynamic characteristic, Bouc–Wen model, particle swarm algorithm, experiment

## Abstract

Electromagnetic spring active isolators have attracted extensive attention in recent years. The standard Bouc–Wen model is widely used to describe hysteretic behavior but cannot accurately describe asymmetric behavior. The standard Bouc–Wen model is improved to better describe the dynamic characteristic of a toothed electromagnetic spring. The hysteresis model of toothed electromagnetic spring is established by adding mass, damping, and asymmetric correction terms with direction. Subsequently, the particle swarm optimization algorithm is used to identify the parameters of the established model, and the results are compared with those obtained from the experiment. The results show that the current has a significant impact on the dynamic curve. When the current increases from 0.5 A to 2.0 A, the electromagnetic force sharply increases from 49 N to 534 N. Under different excitations and currents, the residual points predicted by the model proposed in this work fall basically in the horizontal band region of −20–20 N (for an applied current of 1.0 A) and −40–80 N (for an application of 4.5 mm/s). Furthermore, the maximum relative error of the model is 12.75%. The *R*^2^ of the model is higher than 0.98 and the highest value is 0.9993, proving the accuracy of the established model.

## 1. Introduction

Vibration is a common physical phenomenon in engineering and technology, affecting the operation of the equipment and the mechanical power system while reducing its lifespan [1,2]. The traditional passive vibration isolation system has been widely used in vibration isolation due to its simple structure and low cost [3,4,5]. However, traditional vibration isolation systems cannot be adjusted in real time based on changes in external excitation frequency. With the high-precision requirements related to precision manufacturing and measurement and the demand for environmental noise reduction, traditional passive systems can hardly meet the increasingly strict vibration control requirements [6]. As an active vibration isolation system, electromagnetic springs have the characteristics of fast response, non-contact, and adjustable stiffness [7,8], indicating that it has broad application prospects, especially in the reduction in the vibration of marine engines and air compressors [9,10,11].

Researchers have studied many types of electromagnetic springs and their models. Batdorff et al. [12] proposed a method for calculating multiple-edge magnetic conductance and magnetic leakage in axisymmetric electromagnetic devices. Moreover, the authors established an analytical model of electromagnetic force using the equivalent magnetic circuit method, significantly improving the model’s accuracy. Ertuğrul et al. [13] proposed a new segmented magnetic equivalent circuit method to analyze the magnetic force of the hybrid electromagnet system. In addition, the force characteristics and magnetic field distribution were studied and the results were compared with finite element analysis to verify the effectiveness of the method. Sun et al. [14] constructed a new type of electromagnetic negative stiffness spring using a coaxial permanent magnet ring magnet and rectangular cross-section coil. Furthermore, they established an analytical model based on the filament method to quantitatively study the factors affecting electromagnetic force and stiffness characteristics. Wu et al. [15] established the stiffness analytical model of negative stiffness array magnetic spring based on the magnetic charge model and validated the results via static experiments. Li et al. [16] proposed an improved current filament method, which uses the equivalent circuit principle and considers the skin effect to improve the model’s accuracy by calculating the current of the electromagnetic forming coil and the electromagnetic force of the workpiece. Wang et al. [17] conducted in-depth research on the multi-gap permanent magnet-biased axial magnetic bearing, solved the magnetic leakage of the magnetic bearing using Laplace’s equation and established an accurate analytical model.

The Bouc–Wen model is widely used to represent the properties of materials. The model has an efficient shape control flexibility and was proposed to describe highly asymmetric hysteresis [18,19,20]. In this work, based on the standard Bouc–Wen model, the hysteresis model of a toothed electromagnetic spring is established by introducing the mass, damping, and asymmetric correction terms, while the accuracy of the model is proven by experiments.

## 2. Structure and Principle of a Toothed Electromagnetic Spring

**Figure 1:**
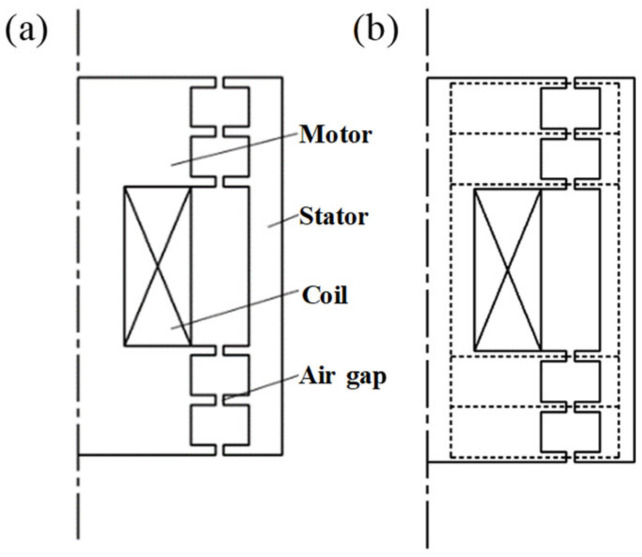
Structure and magnetic circuit of the electromagnetic spring: (**a**) schematic diagram of the toothed electromagnetic spring structure and (**b**) schematic diagram of the magnetic field.

A toothed electromagnetic spring has an axisymmetric structure mainly composed of a motor, stator, coil, and air gap, as shown in Figure 1. The rotor and stator of the toothed electromagnetic spring are arranged with annular teeth of the same size with an air gap between each pair of teeth. The coil is usually wound around the actuator. When the current flows into the coil, the actuator generates an electromagnetic field and flows into the stator’s tooth ring through the air gap. Then, the field flows back into the rotor through the air gap, forming a closed loop.

**Figure 2:**
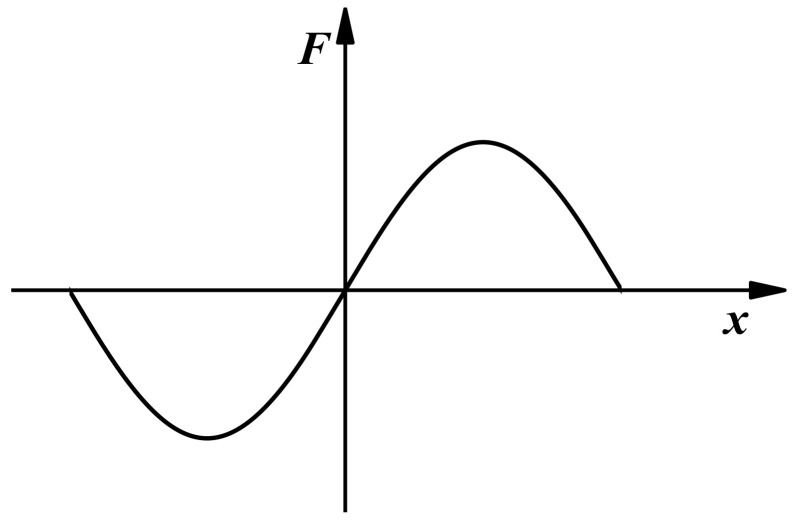
Electromagnetic force characteristics of a toothed electromagnetic spring.

The rotor and the stator of the toothed electromagnetic spring are usually processed with high-permeability materials. At the same time, the air permeability is much lower than that of the rotor and stator. Therefore, the magnetic circuit of the electromagnetic spring is consistent with the direction along the dotted line in Figure 1b, and there is rarely magnetic leakage. Figure 2 shows the relationship between the axial displacement of the rotor (*x*) and the electromagnetic force generated by the toothed electromagnetic spring (*F*). When the axial displacement of the rotor is zero, the electromagnetic spring is in equilibrium. Consequently, the magnetic teeth of each rotor and stator correspond to each other. The electromagnetic force generated between the magnetic teeth only exists in the radial direction, and the resultant force is zero. When the rotor produces axial displacement, there is a displacement deviation between the rotor’s magnetic teeth and the stator’s. At this time, an electromagnetic force is generated between the magnetic teeth, which is approximately proportional to the axial displacement of the rotor within a certain range. Therefore, the electromagnetic force generated by the electromagnetic spring can be adjusted by modifying the coil current, ultimately adjusting the stiffness characteristics of the electromagnetic spring.

## 3. Establishing the Model

### 3.1. Standard Bouc–Wen Model

The standard Bouc–Wen model was first proposed by Bouc [21] to characterize the hysteretic characteristics of materials. Then, the model was further extended by Wen [22]. The standard Bouc–Wen model has been widely used to describe hysteretic nonlinear models, such as piezoelectric actuators and magnetorheological dampers [23,24].

**Figure 3:**
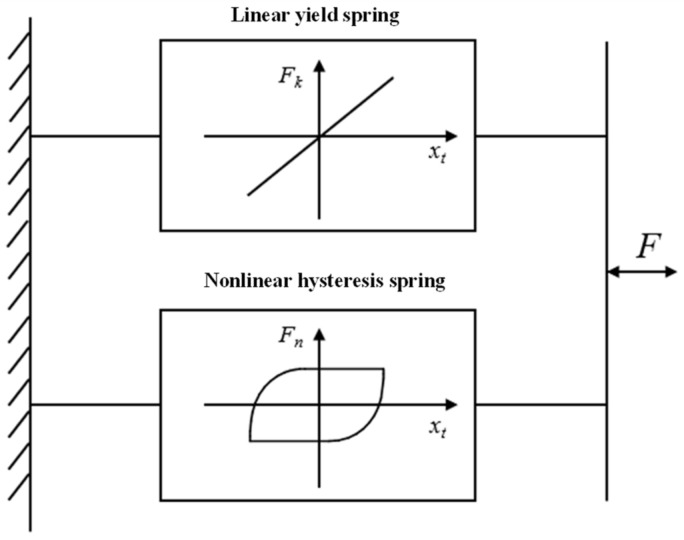
The standard Bouc–Wen model.

The standard Bouc–Wen model can be decomposed into linear yield and nonlinear hysteresis springs connected in parallel, as illustrated in Figure 3. The hysteresis characteristics of this model can be described through a first-order differential equation, as shown in Equation (1) [25]:

(1) $$\left\{\begin{array}{l} F(t)=F_{k}+F_{n}=\alpha \frac{F_{y}}{u_{y}}x\left(t\right)+\left(1-\alpha \right)F_{y}Z\\ \frac{dZ}{dt}=v\left\{A-\left[\gamma +\beta \mathrm{sgn}\left(\dot{x}Z\right)\right]{\left|Z\right|}^{n}\right\} \end{array}\right.$$

where *F* represents the restoring force of the system, *F_k_* represents the linear spring-restoring force, *F_n_* represents the nonlinear hysteretic-restoring force, *F_y_* represents the yield force, *u_y_* represents the yield displacement, *x* represents the relative displacement, *Z* represents the dimensionless hysteretic variable, *sgn*() represents the symbolic function, $\dot{x}$ represents the relative velocity, and *A*, *γ*, and *β* are the shape parameters of the standard Bouc–Wen model.

However, the standard Bouc–Wen model has many parameters, resulting in complex mathematical expressions. Therefore, Vincenzo [26] derived a standard model, which is more concise and conducive to studying parameter identification.

The standard Bouc–Wen model can be expressed as follows:

(2) $$\frac{dz}{dt}=\rho \left[v-\sigma \left|\dot{x}\right|{\left|z\right|}^{n-1}z+\left(\sigma -1\right)\dot{x}{\left|z\right|}^{n}\right]$$

where ρ and σ are the shape parameters of the Bouc–Wen model, with $\rho =\frac{A}{u_{y}\varphi }>0$ and $\sigma =\frac{\beta }{\beta +\gamma }>0.5$; $\varphi =\sqrt[{n}]{\frac{A}{\beta +\gamma }}$, n>1, and *z* is a non-observable dimensionless hysteretic variable, with $z\in \left[-1,1\right]$.

**Figure 4:**
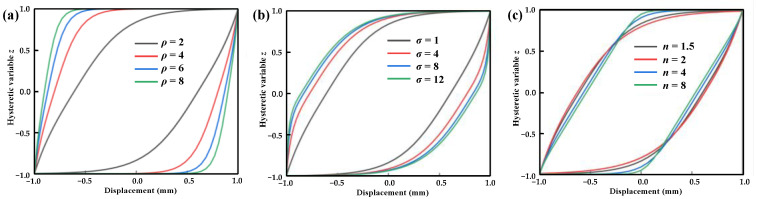
Effect of standard parameters on the hysteresis curve: (**a**) effect of parameter *ρ* on the hysteresis curve (*σ* = 1, *n* = 2), (**b**) effect of parameter *σ* on the hysteresis curve (*ρ* = 2, *n* = 2), and (**c**) effect of parameter *n* on the hysteresis curve (*σ* = 1, *ρ* = 2).

This section aims to explore the impact of these parameters on the hysteresis curve of the system by adjusting the shape parameters of the standard Bouc–Wen model. The performance curve of Equation (2) is shown in Figure 4 by changing the parameters *ρ*, *σ*, and *n*. Figure 4a shows the hysteresis curve under different values of parameter *ρ*. It can be seen that *ρ* mainly changes the hysteresis characteristic of the hysteresis curve at the end of the forward and reverse motion. The higher the *ρ*, the more obvious the hysteresis phenomenon. Similar to parameter *ρ*, a higher value of *σ* increases the hysteresis area, while the influence is not so obvious when *σ* > 8. The parameter *n* mainly influences the bending shape of the hysteresis curve. With a smaller value of *n*, the bending radius of the curve increases, and the curve transition becomes smoother.

### 3.2. Improved Bouc–Wen Model

The hysteresis curve produces different magnitudes when the electromagnetic spring actuator moves forward and backward; therefore, the model exhibits asymmetry. Thus, the standard Bouc–Wen models cannot accurately describe its asymmetric hysteresis characteristics. Hence, it is necessary to improve the Bouc–Wen model.

The Bouc–Wen model shape control function is segmented based on the movement direction of the toothed electromagnetic spring to produce differences in the hysteresis curves of different movement directions, as shown in Equation (3):

(3) $$\rho =\left|\lambda_{1}\mathrm{sgn}\left(v\right)+\lambda_{2}\right|$$

The dynamic forces of electromagnetic springs are divided into linear and hysteresis parts due to the nonlinearity of both static and dynamic forces of electromagnetic springs while considering the addition of mass and damping terms. Thus, Equation (1) can be written as Equation (4):

(4) $$\left\{\begin{array}{l} F=k_{1}z+k_{2}x+cx+m\ddot{x}\\ \frac{dz}{dt}=\left|\lambda_{1}\mathrm{sgn}\left(\dot{x}\right)+\lambda_{2}\right|\left[v-\sigma \left|\dot{x}\right|{\left|z\right|}^{n-1}z+\left(\sigma -1\right)\dot{x}{\left|z\right|}^{n}\right] \end{array}\right.$$

where *k*_1_ is the nonlinear stiffness of the electromagnetic spring; *k*_2_ is the linear stiffness; *λ*_1_, *λ_2_, σ*, and *n* are the shape parameters of the improved Bouc–Wen model; *m* is the mass of the mover; and *c* is the system damping.

**Figure 5:**
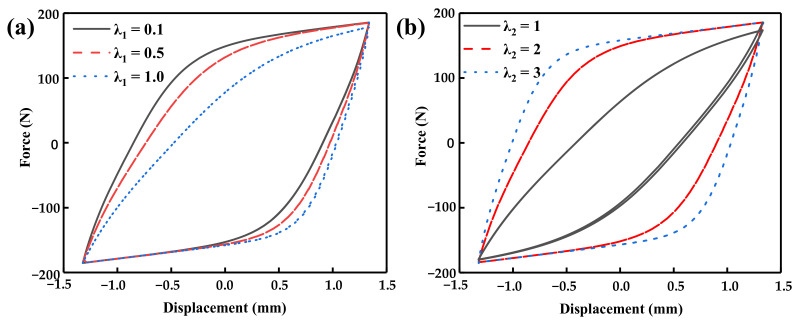
Hysteresis curves for different parameters: (**a**) $\lambda_{1}$ and (**b**) $\lambda_{2}$.

The experimental data at a current of 1.5 A were taken to study the effect of changes in parameters *λ*_1_ and *λ*_2_ on the dynamic characteristics of the toothed electromagnetic spring, and the results are shown in Figure 5.

The experimental results of changing the parameter *λ*_1_ (*λ*_2_ *=* 2) and changing the parameter *λ*_2_ (*λ*_1_ *=* 0.5) are shown in Figure 5a,b, respectively. Figure 5 shows that, the larger λ_1_ is, the more pronounced the hysteresis curve is in asymmetry. As λ_2_ increases, the hysteresis phenomenon of the entire model becomes more pronounced, but the degree of asymmetry decreases accordingly.

### 3.3. Parameter Identification Method

**Figure 6:**
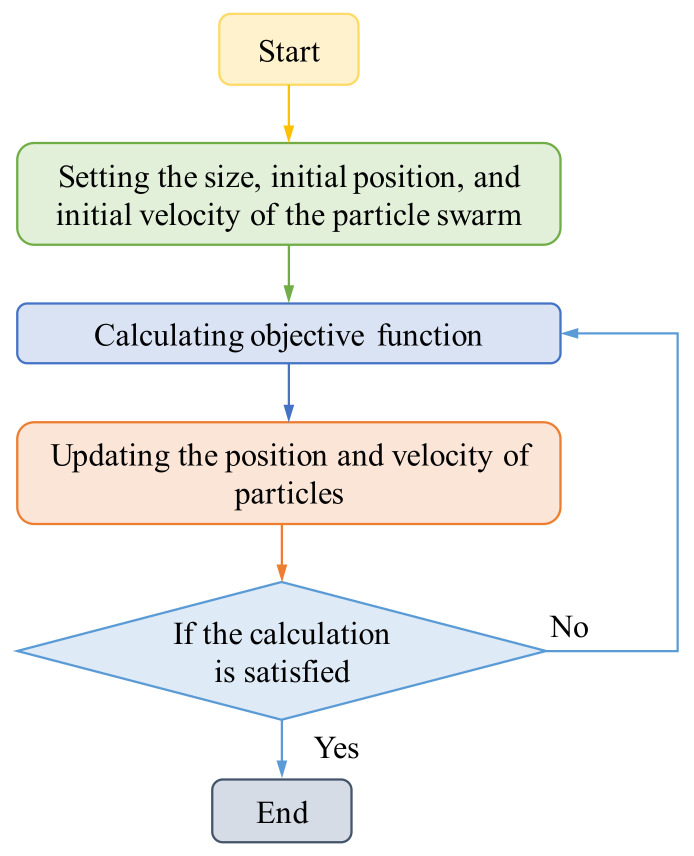
Flowchart of the particle swarm optimization algorithm.

After the dynamic output force and displacement data of the toothed electromagnetic spring were experimentally measured, the dynamic output force and displacement were simulated by the Bouc–Wen model, and the parameters were identified by the algorithm. The commonly used parameter identification algorithms for hysteresis models include the least square method, artificial neural networks, and particle swarm optimization. The least squares method requires multiple iterations to solve the nonlinear models, and the calculation is relatively complex. The artificial neural network method requires a large amount of training data, and the grid parameters must also be adjusted multiple times. The particle swarm optimization algorithm can perform a global search in parameter space and adaptively adjust the search direction and range, making it widely used in parameter recognition. Therefore, the particle swarm optimization algorithm was used to identify the hysteresis model parameters of the toothed electromagnetic spring, and the flowchart is shown in Figure 6.

According to Equation (4), the model has six unknown parameters. Due to differences in the Bouc–Wen model of the toothed electromagnetic spring, the model does not have a true solution, and it cannot be determined whether the solution is optimal. Therefore, the velocity and position of particles were updated according to Equation (5):

(5) $$\left\{\begin{array}{l} x_{i}^{k+1}=x_{i}^{k}+x_{i}^{k+1}\\ \;\;\;\;w=w_{\max}-\frac{(w_{\max}-w_{\min})k}{k_{\max}}\\ v_{i}^{k+1}=wv_{i}^{k}+c_{1}r_{1}(p_{i}-x_{i}^{k})+c_{2}r_{2}(p_{g}-x_{i}^{k}) \end{array}\right.$$

where *p*_i_ is the best position for oneself, *p*_g_ is the best position for the entire population, w is the inertia weight, *c*_1_ and *c*_2_ are learning factors, *r*_1_ and *r*_2_ are random numbers, *v*_i_ is particle velocity, *x*_i_ is the particle position, *k* is the current iteration number, *k*_max_ is the maximum iteration number, *v*_max_ is the inertia weight at the beginning of the iteration, and *v*_min_ is the weight at the end of the iteration.

A fitness function needs to be used to evaluate the advantages and disadvantages of the solution. In this work, the root-mean-square error between the test value of the electromagnetic spring output force and the calculation result was taken as the fitness function, as shown in Equation (6).

(6) $$f_{t}=\sqrt{\frac{\sum_{i=1}^{N_{t}}{\left(F-F_{t}\right)}^{2}}{N_{t}}}$$

where *N_t_* is the total number of samples; and *F_t_* and *F* are the measured and calculated values of the output force of the toothed electromagnetic spring, respectively.

## 4. Experimental Validation

### 4.1. Experimental System and Results

**Figure 7:**
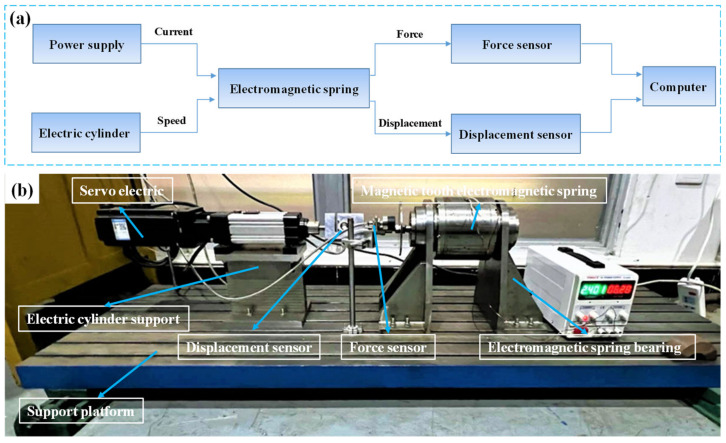
The toothed electromagnetic spring of (**a**) the test flow chart and (**b**) test platform.

The test flowchart and platform of the toothed electromagnetic spring are shown in Figure 7. The DC power supply outputs different currents (or electric cylinder produce different moving speeds), so the electromagnetic spring receives current (or speed) pulses and produces electromagnetic force and displacement. Then, the force sensor and the displacement sensor collect the force and displacement signals, respectively, and send them to the computer for signal processing. During the test, the displacement of the actuator of the electromagnetic spring was adjusted to 1.3 mm, and a constant current was applied to the coil. The electric cylinder was controlled to move at a constant speed until the actuator moved to −1.3 mm. Then, the electric cylinder was controlled to move in the opposite direction at the same speed until the displacement of the actuator reached 1.3 mm. The main technical specifications for the experimental system are illustrated in Table 1. Additionally, a 1 mm polyester imide enameled wire was selected as the solenoid coil in this experiment, with a safe current of 2.75 A and 390 turns.

**Figure 8:**
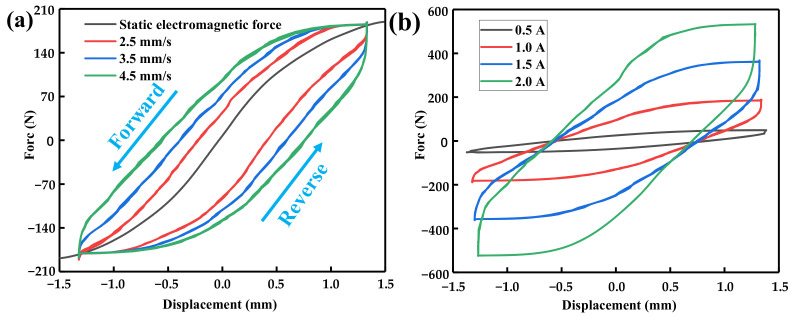
(**a**) Dynamic output force–displacement curve at the current of 1.0 A and (**b**) output force–displacement curve at different currents at the speed of 4.5 mm/s.

Figure 8 shows a significant hysteresis phenomenon between the output force and displacement of the toothed electromagnetic spring. According to Figure 8a, the force–displacement curve exhibits asymmetry when the electromagnetic spring actuator moves in the forward and opposite directions. In addition, under the same current, when the velocity of the mover changes, a significant change in its hysteresis phenomenon is also observed. With the decrease in the moving speed of the actuator, the output force–displacement hysteresis of the electromagnetic spring decreases significantly, and the parallel part of the hysteresis curve with the static curve increases. When the velocity of the actuator is small enough, it can be assumed that the dynamic electromagnetic force characteristics of the electromagnetic spring are the same as those of the static electromagnetic force characteristics. Figure 8b shows the output force–displacement characteristics of the toothed electromagnetic spring under different currents at a rotor speed of 4.5 mm/s.

According to Figure 8b, the hysteresis of the electromagnetic spring gradually increases with the current, i.e., the output force under the same displacement gradually increases. This phenomenon indicates that the electromagnetic spring’s stiffness increases with the current. The increase in the hysteresis also indicates that the nonlinear degree of electromagnetic spring increases gradually during dynamic works. Therefore, in practical applications, it is necessary to conduct reasonable current control according to specific requirements to provide the required output characteristics.

### 4.2. Model Verification

The parameters in the Bouc–Wen model change due to different hysteresis characteristics. In this work, the output force test data generated under different current and speed excitation conditions were selected as *F_t_*. The particle swarm optimization was used to identify the parameters of the improved Bouc–Wen model, in which $w_{\max}=0.9,\;w_{\min}=0.4,\;c_{1int}=2,\;c_{2int}=2,\;c_{1fin}=4,\;\mathrm{and}\;c_{2fin}=4.5$ were considered. Additionally, the number of population particles was set to 100. The maximum number of iterations was 200, and the range of values for each parameter of the Bouc–Wen model is shown in Table 2.

**Figure 9:**
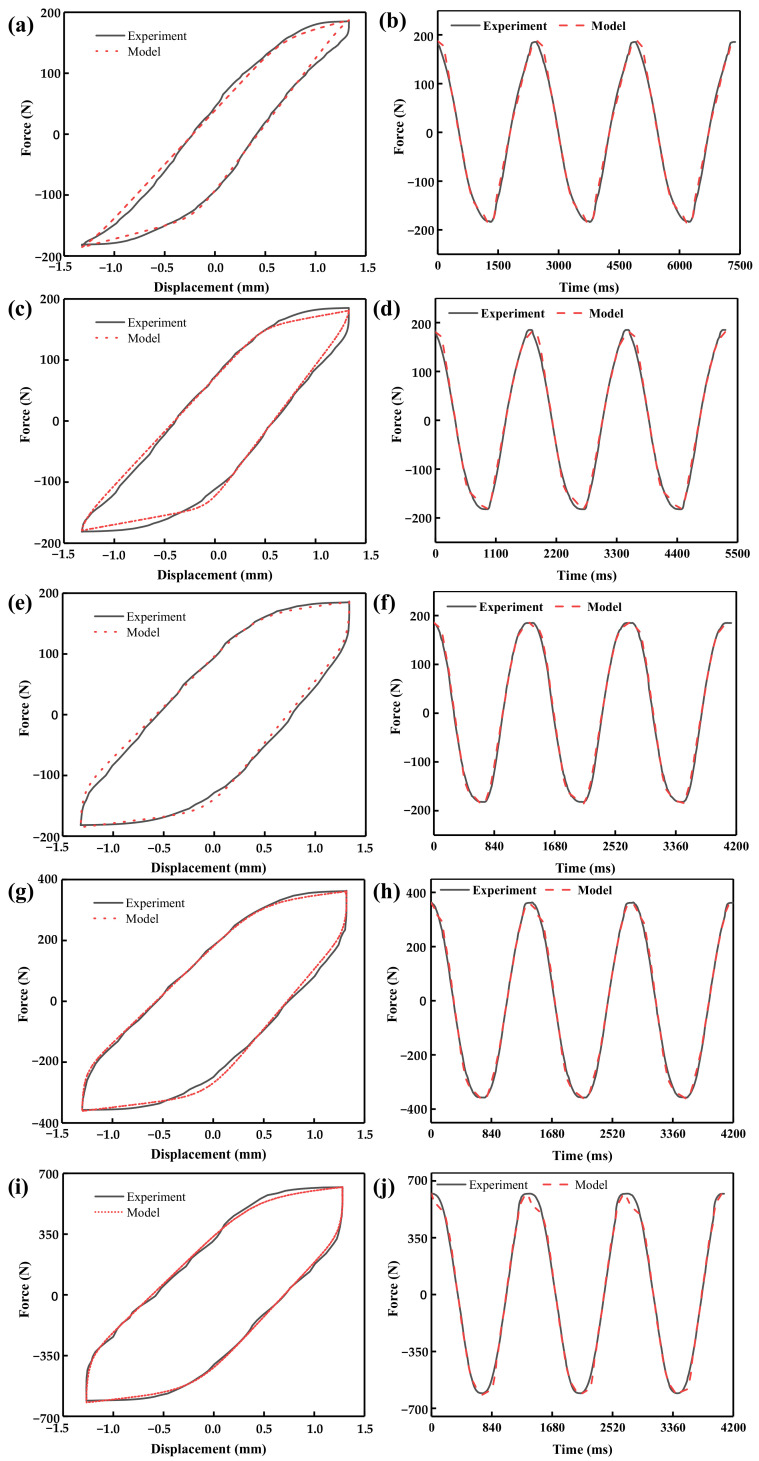
Comparison of the hysteresis characteristics of the toothed electromagnetic spring: (**a**,**b**) for the applied current of 1.0 A and speed of 2.5 mm/s, (**c**,**d**) for the applied current of 1.0 A and speed of 3.5 mm/s, (**e**,**f**) for the applied current of 1.0 A and speed of 4.5 mm/s, (**g**,**h**) for the applied current of 1.5 A and speed of 4.5 mm/s, and (**i**,**j**) for the applied current of 2.0 A and speed of 4.5 mm/s.

Table 3 shows the parameter identification results of the improved Bouc–Wen model using particle swarm optimization. The hysteresis loop of the restoring force of the toothed electromagnetic spring can be reconstructed according to the identification results. A comparison between the estimated curves fitted by the proposed model and experimentally measured results was performed to verify the accuracy of the established model, as illustrated in Figure 9, which shows that the experimental curve is highly consistent with the predicted curve. Therefore, the improved Bouc–Wen model established in this work can accurately describe toothed electromagnetic springs’ dynamic output force–displacement characteristics.

**Figure 10:**
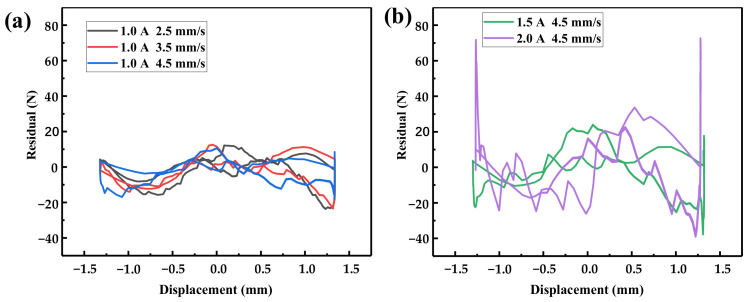
Residual analysis of (**a**) for the applied current of 1.0 A and (**b**) for the application of 4.5 mm/s.

It is necessary to analyze the model error additionally. A residual analysis, which indicates the difference between the measured and predicted results, was adopted to evaluate the prediction accuracy. The results of the residual analysis are shown in Figure 10. It can be seen in Figure 10 that the residual points predicted by the model proposed in this work and the actual experimental results fell basically in the horizontal band region of –20 N–20 N (for an applied current of 1.0 A) and –40 N–80 N (for an application of 4.5 mm/s). This indicates that the constitutive model developed in this work can effectively reflect the dynamic output force–displacement characteristics.

To provide a more quantitative explanation, the maximum relative error of the improved model was defined as follows:

(7) $$\delta_{MAX}=\frac{\Delta F_{MAX}}{F_{MAX}}\times 100\%$$

where *F_MAX_* is the maximum output force measured in the experiment and ∆*F_MAX_* is the maximum output force error of the model compared to the experiment. Meanwhile, *R*^2^ was defined to test the goodness of fit of the improved model, as shown in Equation (7):

(8) $$R^{2}=1-\frac{\sum_{i=1}^{n}{\left({\hat{F}}_{i}-\bar{F}\right)}^{2}}{\sum_{i=1}^{n}{\left(F_{i}-\bar{F}\right)}^{2}}$$

where *F_i_* is the dynamic output force measured in the experiment, $\bar{F}$ is the average value of the dynamic output force, and $\hat{F_{i}}$ is the dynamic output force obtained through model simulation fitting. The correlation index *R*^2^ was used to describe the fitting degree of the model; the closer *R*^2^ is to 1, the better the prediction accuracy of the improved model, and the stronger the linear correlation between the actual variables and the predicted variables [27].

Table 4 illustrates the error results under different excitation currents and speeds. It can be seen that the maximum relative error of the standard Bouc–Wen model was 12.75%. Generally, a Bouc–Wen model with a maximum relative error of less than 15% is considered to have a high accuracy [28]. In addition, the correlation index *R*^2^ of the Bouc–Wen model was higher than 0.98 under different working conditions. The highest value was 0.9993, indicating that the model used in this work has a good fitting accuracy and verifies the accuracy of the model.

## 5. Conclusions

In this work, the standard Bouc–Wen model was improved, and the hysteresis model of a toothed electromagnetic spring was established. Then, the particle swarm optimization algorithm was used to identify the model’s parameters. The main conclusions are as follows:

(1) The influence of various parameters of the standard Bou–Wen model was studied. The results show that the higher the parameter *ρ*, the more serious the hysteresis phenomenon, and the greater the coincidence range between the hysteresis curve and the upper and lower boundary of the hysteresis variable. The higher the *σ*, the larger the coincidence range between the hysteresis curve and the boundary. The higher the parameter *n*, the smaller the bending radius of the hysteresis curve.
(2) The electromagnetic spring exhibits a hysteresis phenomenon under different currents and velocities. The hysteresis phenomenon becomes more pronounced as the speed increases. The current has a significant impact on the dynamic curve. When the current increases from 0.5 A to 2.0 A, the electromagnetic force sharply increases from 49 N to 534 N.
(3) An asymmetric correction term was proposed based on the asymmetry of the dynamic characteristics to replace the parameter *ρ* and improve the standard Bou–Wen model. Compared with the experimental results, the residual points predicted by the model proposed in this work fell basically in the horizontal band region of –20 N–20 N (for an applied current of 1.0 A) and –40 N–80 N (for an application of 4.5 mm/s) and the maximum relative error of the model was 12.75%. The *R*^2^ of the model was greater than 0.98 and the highest value was 0.9993, indicating that the model has a good fitting accuracy.

## Figures and Tables

**Table 1:** The main technical specifications for the experimental system.

Equipment	Model	Parameters	Manufacture
Force sensor	AR-DN23	Range: 0–5 kN Accuracy: 0.015%F.S	Ailixun, Chian
Displacement sensor	ML33-12.5-A	Range: 0–12.5 mm Accuracy: 0.1 %F.S	Miran, China
Servo electric	ECMA-C200807SS	Output: 3000 rpm	Delta, China
Power supply	DC-3010D	Range: 0–10 A, 0–30 V	Yihua, China

**Table 2:** Range of the model parameter values of the Bouc–Wen.

Parameters	Value
σ	(0.5, 100]
n	(1, 20]
$\lambda_{1}$	[0, 10]
$\lambda_{2}$	(0, 10]
$k_{1}$	(0, 1000]
$k_{2}$	(0, 1000]
c	(−∞, +∞)

**Table 3:** Identification results of the particle swarm optimization algorithm.

Current (A)	Speed (mm/s)	*σ*	*n*	*λ*_1_	*λ*_2_	*k*_1_ (N)	*k*_2_ (N/mm)	*c* (N·s/m)
1.0	2.5	0.25	8.13	0.15	1.19	130.69	42.34	589.90
1.0	3.5	1.91	9.98	0.15	1.19	140.59	29.96	611.74
1.0	4.5	18.74	7.03	0.12	1.04	158.37	20.35	646.39
1.5	4.5	21.19	6.59	0.12	1.03	308.78	39.93	654.99
2.0	4.5	20.53	5.42	0.06	0.97	541.70	62.31	665.09

**Table 4:** Error analysis of the Bouc–Wen model.

Current (A)	Speed (mm/s)	∆*F_MAX_* (N)	*F_MAX_*	*δ_MAX_*	*R* ^2^
1.0	2.5	23.65	185.49	12.75%	0.9991
1.0	3.5	23.46	185.49	12.68%	0.9959
1.0	4.5	18.04	185.49	9.68%	0.9971
1.5	4.5	37.84	363.49	10.41%	0.9993
2.0	4.5	72.65	621.47	11.69%	0.9868

## Data Availability

The data presented in this study are available upon request from the corresponding author. The data are not publicly available due to privacy.
